# Metabolic impact of low dose IL-2 therapy for primary Sjögren’s Syndrome in a double-blind, randomized clinical trial

**DOI:** 10.1007/s10067-024-07165-2

**Published:** 2024-10-31

**Authors:** Ruiling Feng, Xian Xiao, Yifan Wang, Bo Huang, Jiali Chen, Gong Cheng, Yuebo Jin

**Affiliations:** Department of Rheumatology and Immunology, Beijing Key Laboratory for Rheumatism Mechanism and Immune Diagnosis (BZ0135), Peking University People’s Hospital, 11 Xizhimen South St., Beijing, 100044 China

**Keywords:** Low-dose interleukin 2 therapy, Primary Sjögren’s syndrome, Profile hydrophilic metabolites, T helper cell, Treg

## Abstract

**Objectives:**

Low-dose interleukin 2 (Ld-IL2) is increasingly being explored as an immune-modulating treatment for autoimmune diseases which mainly affect T cell subsets. This study investigates the metabolic effects of Ld-IL2 therapy in patients with primary Sjögren’s syndrome (pSS).

**Method:**

A total of 60 patients were recruited to conduct a double-blind, randomized clinical trial. Of these patients, 50% (30/60) received Ld-IL2 therapy along with standard treatment for 12 weeks, followed by 12 weeks of follow-up. The effectiveness was evaluated by Sjögren's Tool for Assessing Response (STAR). An untargeted analysis was performed to profile hydrophilic metabolites.

**Results:**

Metabolic profiling revealed significant alterations post-treatment, notably in metabolites like acetyl-CoA, ascorbic acid, and glutathione, which are beneficial in managing autoimmune diseases. In addition, the levels of metabolite accumulation were correlated with variations in immune cell subsets (*p *< 0.05), particularly Tregs. Moreover, patients exhibiting a specific metabolic profile, including lower serum levels of isoleucine, ADP, Thymidine 5'-triphosphate, and other metabolites, had a high response rate (91.7%-98.6%), as indicated by the receiver operating characteristic (ROC) curve.

**Conclusions:**

These findings suggest that Ld-IL2 therapy influences metabolic pathways in pSS, offering insights into the systemic effects of Ld-IL2 therapy beyond immune modulation.

**Trial registration number:**

ClinicalTrials.gov number, NCT02464319.

**Table:** 

**Key Points** • *Metabolic alteration in pSS is significantly associated with Ld-IL2 therapy.* • *Metabolic changes correlate with variations in immune cell subsets, particularly Tregs.* • *Metabolic profiling could be a valuable tool in guiding Ld-IL2 therapy choices for pSS patients.*

**Supplementary Information:**

The online version contains supplementary material available at 10.1007/s10067-024-07165-2.

## Introduction

Primary Sjögren's Syndrome (pSS) is an autoimmune disorder characterized by the dysfunction and destruction of exocrine glands, predominantly salivary and lacrimal glands, leading to symptoms such as dry mouth and dry eyes. The etiology of pSS is complicated, and one of its hallmarks is the dysregulation of the immune system, particularly the imbalance in T cell subsets, including regulatory T cells (Tregs).

In recent years, Low-dose interleukin 2 (Ld-IL2) therapy has emerged as a promising treatment for a range of autoimmune diseases, including systemic lupus erythematosus (SLE) [1], rheumatoid arthritis (RA) [2], and dermatomyositis (DM) [3]. Recently, we have conducted a randomized, controlled study in which we specifically highlighted the effectiveness and tolerability of Ld-IL2 in treating pSS [4]. Our previous work and other studies have established the potential of Ld-IL2 in modulating immune cells, particularly in enhancing Tregs while suppressing the pro-inflammatory subsets [5–8]. However, the broader implications of Ld-IL2, especially concerning metabolic regulation, remain underexplored.

Emerging evidence suggests that the dysregulation of metabolism functionally contributes to the development and differentiation of immune cells [9, 10]. Advances in our capacity to analyze metabolism in diseased tissues have sparked significant interest in understanding the impact of cellular metabolic status on immune responses. In response to pro-inflammatory stimuli, myeloid and lymphoid cells undergo a metabolic switch towards increased aerobic glycolysis, which regulates the balance between inflammatory and regulatory immune homeostasis [11, 12]. Simultaneously, classically activated macrophages and effector lymphocytes such as T helper 1 (Th1) and T helper 17 (Th17) cells exhibit active glycolysis. However, robust oxidative metabolism favors the differentiation of Treg cells [13–16]. Metabolic dysregulation can result in cellular transformation, autoimmunity, and metabolic disorders [10]. Therapies targeting metabolism modulation toward immunoregulatory pathways hold promise in enhancing the homeostasis of immune cell subsets and thereby mitigating inflammation [17].

This study aims to elucidate the metabolic changes following Ld-IL2 treatment in patients with pSS. By analyzing hydrophilic metabolites in pSS patients treated with Ld-IL2, we provide insights into the metabolic changes and their association with clinical outcomes. Such insights are essential for optimizing treatment strategies and improving the quality of life for patients with pSS and potentially other autoimmune diseases.

## Materials and methods

### Participants

We carried out a phase II, randomized, double-blind, placebo-controlled trial with a 2-group, parallel-controlled, superiority design (NCT02464319). A total of 60 pSS patients were enrolled in the clinical trial; 30 patients received Ld-IL2 therapy, and the other 30 received a placebo. Complete details regarding the study design and inclusion/exclusion criteria have been previously published [4]. All active pSS patients who had an inadequate response to standard treatment for ≥3 months were enrolled. IL-2 (1 million IU) was administered subcutaneously every other day for two weeks (seven injections), followed by a 2-week break, constituting one treatment cycle of 4 weeks. All patients in the study underwent treatment for the initial 12 weeks, including three treatment cycles with IL-2. Treatment response was measured by Sjögren's Tool for Assessing Response (STAR) [18]. A STAR response was defined as 5 points or higher after treatment. This study was conducted by the Declaration of Helsinki, the International Conference on Harmonization Guidelines for Good Clinical Practice, and our experimental protocol was approved by the Human Ethics Committee of Peking University People’s Hospital (Beijing, China). Table 1 summarized baseline characteristics of pSS patients. Written informed consents were obtained from these healthy controls.

**Table 1:** Baseline Characteristics of primary SS patients of the Participants

Characteristics	Low-dose IL-2 (*n* = 30)
Age, median (IQR), y	56 (45 to 61)
Female, No. (%)	30 (100)
Weight, median (IQR), kg	59.0 (52.8 to 64.3)
Height, median (IQR), cm	160.0 (157.0 to 164.3)
BSA, median (IQR), m^2^	1.59 (1.48 to 1.68)
Disease Duration, median (IQR), y	4.5 (3.0 to 7.0)
ESSDAI score	8.0 (6.0 to 12.0)
Dryness VAS score	7.0 (7.0-8.0)
Pain VAS score	4.0 (4.0-7.0)
Fatigue VAS score	7.0 (5.0-7.3)
ESSPRI score	6.3 (5.3-7.1)
MFI-20 score	51.5 (41.0 to 62.0)
SF-36 PCS score	50.3 (49.4 to 50.8)
SF-36 MCS score	50.1 (49.3 to 50.5)
Systemic signs, No. (%)	
Parotid gland enlargement	12 (40.0)
Articular	10 (33.3)
Leukopenia	12 (40.0)
Anemia	3 (10.0)
Thrombocytopenia	4 (13.3)
Pulmonary	12 (40.0)
Renal	2 (6.7)
Neurologic	1 (3.3)
Cutaneous	1 (3.3)
IgA, g/L	4.0 (3.0 to 5.1)
IgG, g/L	22.9 (20.7 to 27.6)
IgM, g/L	1.2 (0.9 to 1.5)
Hypergammaglobulinemia, No. (%)	28 (93.3)
ESR, mm/hr	29.0 (19.0 to 40.0)
C3, g/L	1.00 (0.89 to 1.15)
C4, g/L	0.20 (0.14 to 0.25)
Anti Ro/SSA, positive, No. (%)	27 (90.0)
Anti La/SSB, positive, No. (%)	14 (46.7)
ANA, ≥ 1:320 positive, No. (%)	14 (46.7)
RF, positive, No. (%)	24 (80.0)
DLCO, (%)	66.8 (61.2-70.1)
Background medication, No. (%)	
Glucocorticosteroids	1 (3.3)
Prednisone dose, median (range), mg/d	7.5
Hydroxychloroquine	30 (100)
Cyclosporine	0 (0.0)
Tacrolimus	1 (3.3)
Azathioprine	0 (0.0)
Leflunomide	0 (0.0)

*IL-2* intrleukin 2, *BSA* Body surface area, *ESSDAI* European League Against Rheumatism (EULAR) Sjögren’s syndrome disease activity index, *ESSPRI* EULAR Sjögren’s syndrome Patient Reported Index, *MFI-20* Multidimensional Fatigue Inventory-20, *SF-36 PCS* Short Form (36 Items) physical component scores, *SF-36 MCS* Short Form (36 Items) mental component scores, *IgA* immunoglobulin A, *IgG* immunoglobulin G, *IgM* immunoglobulin M, *ESR* erythrocyte sedimentation rate, *C3* Complement 3, *C4* Complement 4, *ANA* Anti-nuclear antibody, *RF* rheumatoid factor. This table included patients originally randomly assigned to IL-2 and placebo

### Metabolic analysis

Peripheral blood was processed for analysis using a gas chromatography-mass spectrometer. Chromatographic analysis used the Agilent Technologies 1260 A GC system with a flame ionization detector (FID). A fused-silica capillary column with a free fatty acid phase (DB-FFAP, 30 m × 0.53 mm × 0.5 um) was used. Hydrogen served as the carrier gas at a flow rate of 14.4 ml/min. An initial temperature of 100°C was held for 0.5 min, then raised to 180 °C at 8 °C/min and held for 1 min. Subsequently, the temperature was increased to 200 °C at 20 °C/min and was finally held at 200 °C for 5 min. The injection volume was 1 μl, and the run time for each analysis was 17.5 min.

Integrated peak areas corresponding to metabolite concentrations were further analyzed using the MetaboAnalyst 6.0 website (http://www.metaboanalyst.ca). Metabolite abundance was expressed relative to the internal standard.

### Immunological analysis

Peripheral blood mononuclear cells (PBMCs) were incubated with fluorophore-conjugated monoclonal antibodies for 30 minutes at room temperature (Supplementary Table 1). Relative proportions of CD4+T subsets were analyzed by flow cytometry using a FACSAria II instrument (BD Biosciences, San Jose, CA, USA) and FlowJo V10.8.1 software (Tree Star, USA). Relative proportions of Treg, Tfh, Th1, Th2, and Th17 cell subsets were analyzed by flow cytometry using FACSAria III (BD Biosciences, San Jose, CA, USA) and FlowJo software (Tree Star, USA). Treg cells were defined as CD3^+^ CD4^+^ CD25^high^ CD127^low^, Tfh cells as CD3^+^ CD4^+^ CD45RA^-^CXCR5^+^ PD1^+^ CCR7^low^, Th1 cells as CD3^+^ CD4^+^ CXCR3^+^ CCR6^-^ CCR4^-^ CCR7^low^, Th2 cells as CD3^+^ CD4^+^ CXCR3^-^ CCR6^-^ CCR4^+^ CCR7^low^ and Th17 cells as CD3^+^ CD4^+^ CXCR3^-^ CCR6^+^ CCR4^+^ CCR7^low^.

### Statistical analysis

Data were expressed as the median and range for non-normally distributed data, while mean ± standard deviation (SD.) for normally distributed data. The student's unpaired or paired t-test was performed to compare two groups for parametric data, and the Mann-Whitney U test or Wilcoxon rank sum test was performed for nonparametric data. Spearman's rank test analyzed relationships between variables. Statistical analyses were performed using SPSS v.22.0 or R v.3.6.3 software. Two-sided *p* values < 0.05 were considered statistically significant.

## Results

### Characteristics of pSS patients

The present study investigated the metabolic disruption caused by Ld-IL2. In a double-blind, randomized cohort of 60 pSS patients, half were assigned to receive Ld-IL2 therapy (*n *= 30), and the other half received placebo (*n* = 30). Table 1 details the demographic and clinical manifestations of patients in the Ld-IL2 group.

### Serum Metabolism alterations after Ld-IL2 therapy

**Fig. 1:**
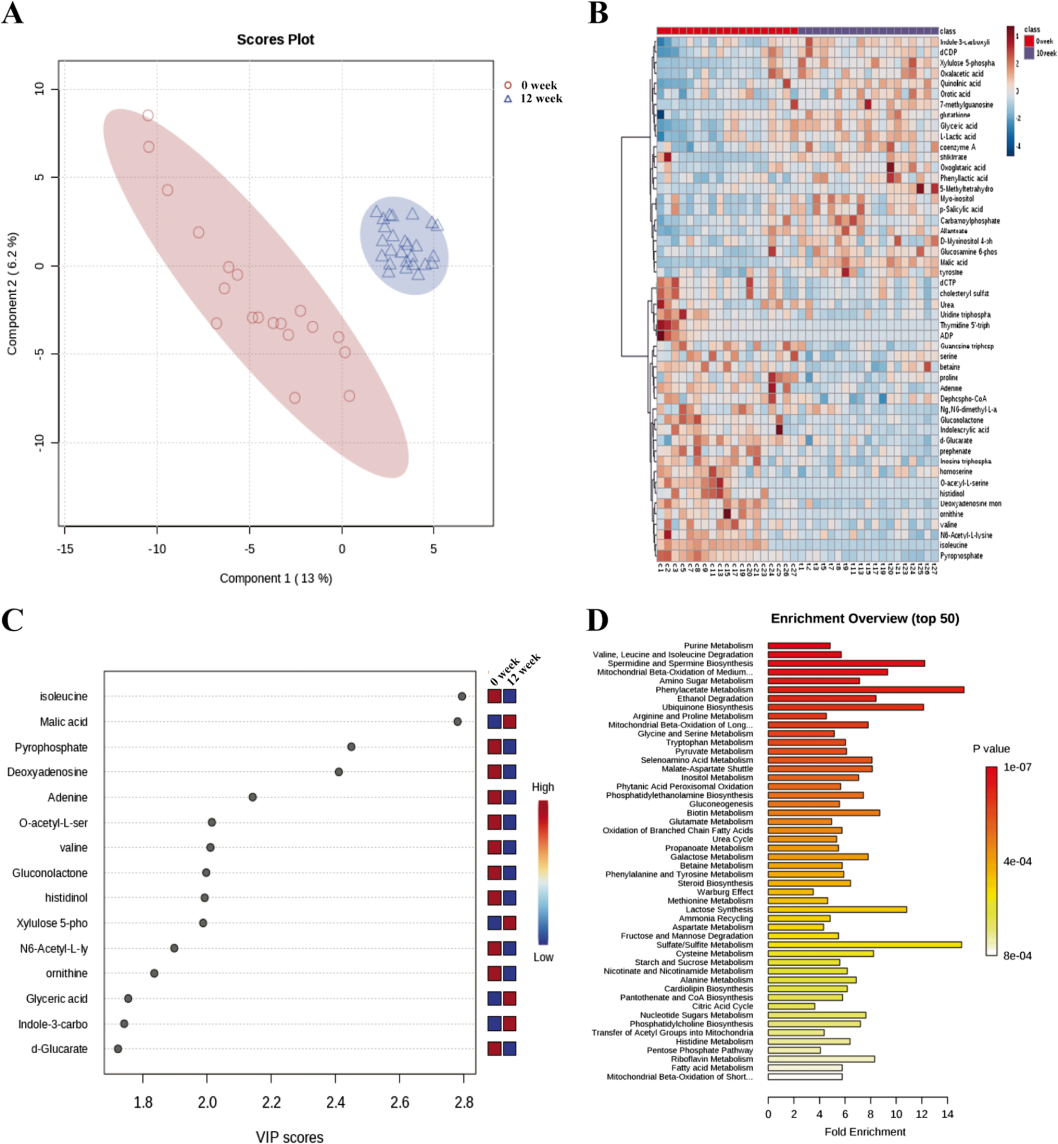
Impact of Ld-IL2 on serum metabolites from pSS patients. (**a**) Partial least-squares-discriminant analysis (PLS-DA) Differentiation depicts the separation of serum metabolite profiles before (0-week, red) and after (10-week, blue) Ld-IL-2 treatment, with PC1 and PC2 explaining 13.9% and 5.5% of variance respectively. A significant distinction between the groups was confirmed (*p* < 0.0001). (**b**) Heat map showing the distinct clustering of pre- and post-treatment groups. (**c**) VIP Scores of PLS-DA showed the important taxa in distinguishing between the two time points (week 0 and week 12 after treatment). A taxon with VIP score >1 was considered important in the discrimination. (**d**) Pathway enrichment analysis elucidates the metabolic pathways affected by Ld-IL2 therapy. LSM, least-squares mean; VAS, visual analogue scale. Global *p* values are based on type III test evaluated with longitudinal regression analyses. *p* value was determined by a two-tailed Wilcoxon matched-pairs signed rank test. All the data are presented as the mean ± s.e.m. *, *p *< 0.05; **, *p *< 0.001, ***, *p *< 0.0001

Principal component analysis (PCA) was employed to observe two distinct clusters of serum metabolites before and after Ld-IL2 administration (Fig. 1a). We performed a targeted analysis to profile bile acids and short fatty acids alongside an untargeted analysis to depict hydrophilic and phospholipid metabolites. Apparent differences in metabolite accumulation before and after Ld-IL2 administration are evident in a heatmap displayed for samples arranged via hierarchical clustering (Fig. 1b). Similarly, partial least squares-discriminant analysis (PLS-DA) plot indicated distinct metabolic profiles before and after Ld-IL2 treatment. VIP scores extracted from the PLS-DA model highlighted the top 15 varied metabolites mainly contributing to the metabolic profile resulting from Ld-IL2 treatment (Fig. 1c).

Metabolic analysis revealed significant increases in the concentration of several metabolites known to be beneficial in the treatment of autoimmune diseases, including acetyl-CoA, ascorbic acid, citrate, butyryl-CoA, glutathione, glycoursodeoxycholic acid (GUDCA), and acetic acid, while significant decreases were observed in isoleucine and urea, among others (Supplementary Table 2). The annotated functional associations of these metabolites are detailed in Supplementary Table 2. Overall, these results indicate an Ld-IL2-driven shift toward a metabolic environment that may support immune regulation and tissue repair.

### Changes of metabolic pathways linked to Ld-IL2 therapy

To identify potentially biologically meaningful patterns affected by Ld-IL2 therapy, we utilized the metabolomics data to perform a pathway enrichment analysis using the KEGG metabolic library and Metaboanalyst 6.0. This analysis revealed notable changes in metabolic pathways in serum samples following IL-2 therapy (Fig. 1d). The affected pathways primarily involve anti-inflammation and oxidative stress mediation, showing significant enrichment in processes related to phenylacetate metabolism, ubiquinone biosynthesis, pyruvate metabolism, glutamate metabolism, urea cycle, and CoA biosynthesis. These pathways have previously been documented to functionally contribute to regulating immune cells (Supplementary Table 3).

### Association between metabolite change and immune cells variations

**Fig. 2:**
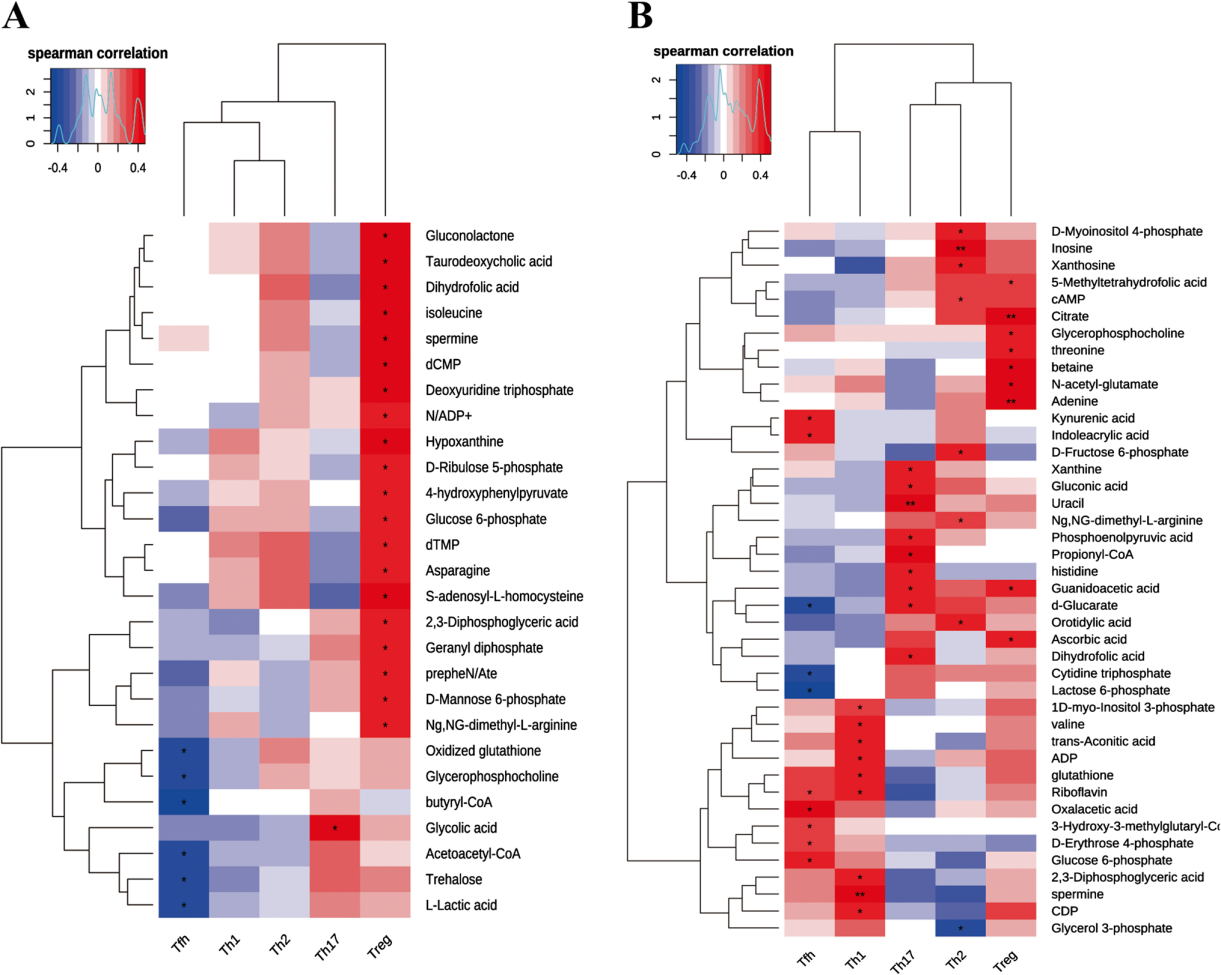
Association between metabolites and CD4+T cells. (**a**) Pre-Treatment Correlation Heat Map showing the Spearman correlations of pSS-associated metabolites with T cell subsets at week 0 (before the IL-2 treatment). (**b**) Post-Treatment Correlation Heat Map showing the association between metabolites and T cell subsets 10 weeks after IL-2 treatment. LSM, least-squares mean; VAS, visual analogue scale. Global *p* values are based on type III test evaluated with longitudinal regression analyses. *p* value was determined by two-tailed Wilcoxon matched-pairs signed rank test

To explore Ld-IL2-driven metabolic changes that might have an impact on immune response regulation, we analyzed the relationship between metabolite accumulation levels and immune cell subset data from the same patients sampled at the same time points (Fig. 2). A significant association between the levels of various metabolites and changes in immune cell subsets were observed, particularly Treg (Fig. 2a and b), but also other T cells, B cells, dendritic cells, and natural killer cells (Supplementary Table 2). As expected, several metabolites that showed significant increases following Ld-IL2 treatment are known to have roles related to adaptive immunity (Supplementary Table 2), indicating that the metabolic shift induced by IL-2 therapy might not only reflect the direct effect of the treatment but could also actively result in these immunomodulatory effects.

### Metabolites Biomarker Identification for Ld-IL2 Treatment Response

**Fig. 3:**
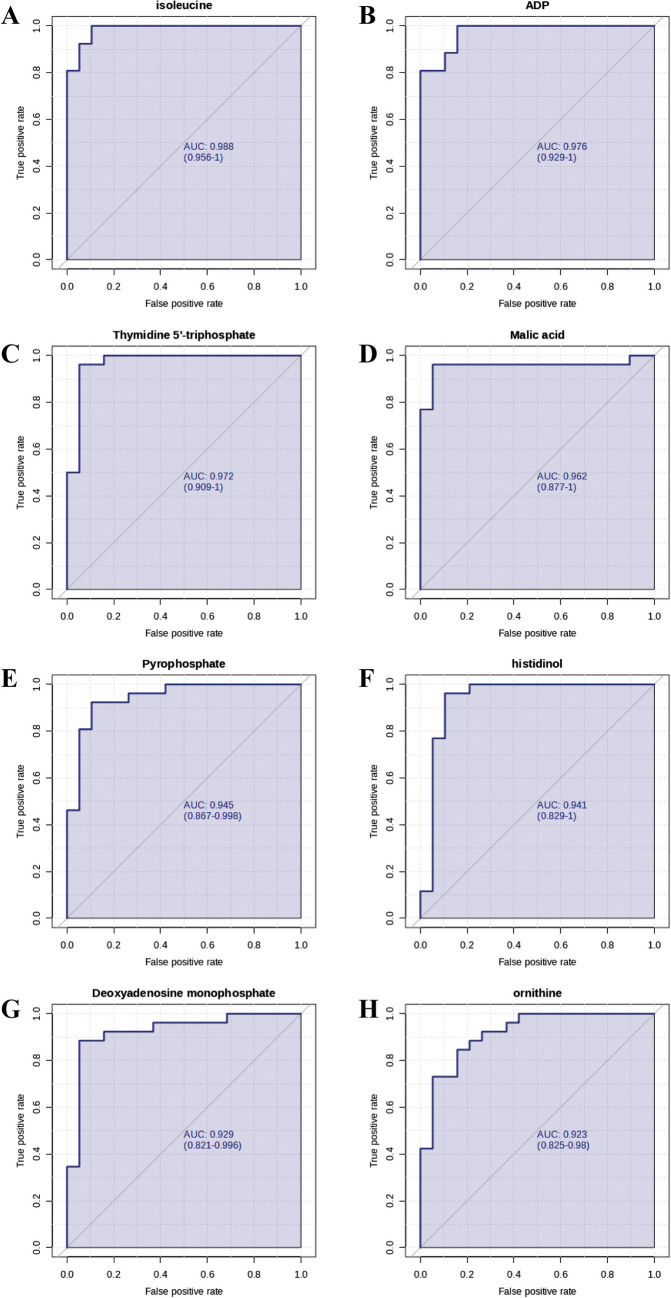
Metabolites Biomarker Identification for Ld-IL2 Treatment Response. (**a-h**) Assesses the diagnostic potential of metabolite response biomarkers in pSS patients by analyzing their sensitivity and specificity through ROC curves. AUC: area under the curve

To identify factors predicting potential response to Ld-IL2 in pSS, we divided the IL-2 arm in the previous cohort into two groups according to Sjögren’s Tool for Assessing Response (STAR). The baseline clinical characteristics of the different groups are shown in Table 2. Patients exhibiting specific metabolic changes showed a response to Ld-IL2. Specifically, lower serum levels of isoleucine, ADP, Thymidine 5'-triphosphate, Malic acid, Pyrophosphate, histidine, Deoxyadenosine monophosphate, and ornithine were associated with a favorable response to Ld-IL2. The response rate ranged between 91.7%-98.6% in these patients (Fig. 3), as determined by the area under the receiver operating characteristic (ROC) curve. It is noted that isoleucine, as a pivotal metabolite involved in the mTOR pathway [19], showed the highest sensitivity and specificity with an area under the curve (AUC) value of 0.986 (Fig. 3a and Table 3).

**Table 2:** The baseline clinical characteristics in response and non-response group

Characteristics	Response (*n *= 19)	Non-response (*n *= 11)	*P* value
Age (years), mean (SD)	50.1 (11.8)	50.7 (13.4)	0.894
Disease duration (years), median (IQR)	3 (2-7)	5 (3-6)	0.578
Disease activity indexes, median (IQR)			
ESSDAI score	3 (3-5)	3.5 (3-5)	0.865
MFI-20 score	66 (59-79)	69 (58, 83)	0.854
Disease parameters, median (IQR)			
WBC, × 10^9^/L, median (range)	3.83 (3.29-5.36)	3.40 (3.15-4.71)	0.449
Lymphocyte, × 10^9^/L, median (range)	1.42 (1.01-1.83)	1.24 (0.94-1.78)	0.542
Platelet, × 10^12^/L, median (range)	200 (169, 262)	188 (137, 231)	0.358
hemoglobin, median (range)	125 (119, 130)	129 (123, 137)	0.124
IgA, g/L, median (range)	3.73 (2.75, 4.91)	4.73 (3.86, 5.97)	0.136
IgG, g/L, median (range)	23.7 (20.8, 30.20)	22.9 (20.80, 25.93)	0.663
IgM, g/L, median (range)	1.26 (1.01, 1.52)	0.97 (0.82, 1.53)	0.142
C3, g/L, median (range)	1.01 (0.88, 1.08)	0.95 (0.87, 1.24)	0.982
C4, g/L, median (range)	0.19 (0.14, 0.23)	0.19 (0.15, 0.30)	0.551
Anti-Ro/SSA, positive, No. (%)	16 (84.2)	10 (90.9)	0.603
Anti-La/SSB, positive, No. (%)	8 (42.1)	7 (63.6)	0.256
ANA, ≥ 1:320 positive, No. (%)	9 (47.4)	7 (63.6)	0.389
RF, positive, No. (%)	17 (89.5)	8 (72.7)	0.236

**Table 3:** Diagnostic efficacy parameters of different metabolites

Metabolites	AUC(95% CI))	Log_2_ (FC)	Sensitivity	Specificity
isoleucine	0.98583	2.6780	100.0%	100.0%
ADP	0.97368	5.7948	84.2%	100.0%
Thymidine 5'-triphosphate	0.96964	4.2974	94.7%	96.2%
Malic acid	0.95547	-1.3105	96.2%	89.5%
Pyrophosphate	0.94332	1.3986	89.5%	96.2%
histidinol	0.93725	4.2987	89.5%	96.2%
Deoxyadenosine monophosphate	0.9251	1.2779	94.7%	92.3%
ornithine	0.917	2.3376	89.5%	84.6%

## Discussion

The findings from our study shed light on the complex interplay between metabolic changes and immune regulation in the context of Ld-IL2 therapy for pSS. In this trial, IL-2 therapy leads to a remarkable increase in concentrations of acetyl-CoA, ascorbic acid, citrate, butyryl-CoA, glutathione, GUDCA, and acetic acid, aligning with research on enriched phenylacetate metabolism, ubiquinone biosynthesis, pyruvate metabolism, glutamate metabolism, urea cycle, and CoA biosynthesis. These Ld-IL2-induced metabolic changes might be pivotal in modulating immune responses, particularly in enhancing the function of regulatory Tregs, which are vital in maintaining immune tolerance.

Currently, there is strong evidence supporting the critical role of metabolism as a driver of immune cell progress and differentiation, participating in both the development and effector functions of diverse immune cells [10, 20]. For instance, isoleucine exerts essential effects on the mTOR (mammalian target of rapamycin) signaling pathway, which is involved in metabolism, cell growth, and immune responses [21]. Similarly, glutamate has been associated with modulating the Th17/Treg balance [22]. In fact, Tao et al*.* (2017) established that inhibiting the metabolic conversion of glutamate into alpha-ketoglutaric acid (α-KG) epigenetically up-regulates FOXP3 expression [23], which consequently blocks Th17 cell differentiation by antagonizing RORγt function, thereby favoring the Treg population. Acetic acid and Butyryl-CoA participate in short-chain fatty acid (SCFA) metabolism [24, 25], elevating the number and function of induced regulatory T cells. Others have illustrated how the glycolytic enzyme glyceraldehyde 3-phosphate dehydrogenase (GAPDH) down-regulates aerobic glycolysis in myeloid and lymphoid cells, preventing immune activation. Additionally, GAPDH can shift the balance between inflammatory and regulatory cell types [11, 26, 27].

In addition to its effects on metabolism and the consecutive autoimmune interaction, IL-2 also altered the energetic state of the patients by promoting acetyl-CoA production after Ld-IL2 treatment. Others have demonstrated that acetyl-CoA acts as a central metabolic intermediate, influencing the activity or specificity of multiple enzymes and reflecting the general energetic state of several immune cell types [28]. Hence, the alteration of the energetic states could explain the significant resolution of fatigue observed in most SS patients after the IL-2 treatment. Simultaneously, this study showed that patients with lower serum levels of metabolites like isoleucine and ADP exhibited higher response rates to Ld-IL2 therapy, suggesting that these metabolic profiles may be linked to milder disease manifestations. Furthermore, our pathway enrichment analysis indicates that key metabolic pathways involved in immune regulation and oxidative stress were significantly altered, which could be tied to the disease's systemic effects, including fatigue and inflammation. These findings suggest a potential connection between the identified metabolic profiles and clinical manifestations such as fatigue and immune dysregulation, characteristic of Sjögren's Syndrome.

Our study is the first to identify that Ld-IL2 can restore both immune and metabolism imbalances, reinforcing the notion that metabolic and immune responses are intricately linked. The correlation between changes in metabolite levels and alterations in immune cell subsets, especially Tregs, highlights the potential of metabolic profiling to monitor and predict the efficacy of IL-2 therapy in pSS patients.

This study limited the response rate of Ld-IL2 therapy in pSS. Thus, optimizing patient stratification would help identify the clinical conditions to determine the individuals that extensively benefit from Ld-IL2 therapy. Identifying specific metabolites that correlate with a favorable response to Ld-IL2 therapy opens new avenues for personalized medicine. Responders of Ld-IL2 therapy were observed to present lower serum citraconic acid, isoleucine, succinate, and higher uridine, among other metabolites. Monitoring changes in key metabolites during therapy may provide clinicians with real-time insights into treatment efficacy, allowing for dynamic adjustments to treatment regimens. This approach could be especially valuable in complex autoimmune diseases such as primary Sjögren’s syndrome, where immune and metabolic dysregulation are intertwined.

However, our study has several limitations that should be addressed in future research. The relatively small sample size limits the generalizability of our findings, and the short duration of follow-up may not capture the long-term effects of the therapy. Follow-up studies to monitor patients over an extended period to assess whether the metabolic changes and clinical benefits observed in this study are sustained. Additionally, our study focused on the effects of low-dose interleukin 2 therapy alone without considering potential interactions with other treatments commonly used in patients with primary Sjögren's Syndrome. Future research should aim to enroll larger, more diverse patient cohorts and explore the impact of combination therapies to provide a more comprehensive understanding of the efficacy and safety of Ld-IL2.

Although our research focused on pSS, the metabolic principles identified herein are applicable to a broader spectrum of autoimmune diseases. This opens up possibilities for novel therapeutic approaches that harness the relationship between metabolic pathways and immune responses and potentially extend the scope of IL-2 therapy. Therefore, further studies on other autoimmune conditions are warranted in the future.

While we observed a correlation between metabolic alterations and changes in immune cell subsets, it is essential to note that our study fails to establish a causal link. Further experimental research is required to clarify whether these metabolic shifts directly result from Ld-IL2 therapy or if they play a more active role in modulating the immune response.

In summary, the study presented here provides preliminary evidence supporting the association between Ld-IL2 therapy and the restoration of metabolic balances. The potential for using metabolic profiling as a predictive tool for therapeutic response is particularly promising, suggesting a future where treatment for autoimmune conditions is more tailored and effective. By exploring specific metabolic pathways and their interplay with immune function, our findings offer novel insights that could inform the clinical application of Ld-IL2 therapy and potentially pave the way for new therapeutic strategies in the management of autoimmune diseases.

## Supplementary Information

Below is the link to the electronic supplementary material.

Supplementary file1 (PDF 158 KB)

## Data Availability

The data that support the findings of this study are available from the corresponding author (Jing He), upon reasonable request.
