# Supplementary material for: Metabolic impact of low dose IL-2 therapy for primary Sjögren’s Syndrome in a double-blind, randomized clinical trial
Source: Clin Rheumatol. 2024 Oct 31;43(12):3789–98. doi: 10.1007/s10067-024-07165-2 (PMC11582071; doi:10.1007/s10067-024-07165-2)
Supplement: Supplementary file 1 — Supplementary file1 (PDF 158 KB) [file 10067_2024_7165_MOESM1_ESM.pdf]

Supplementary Appendix

This appendix has been provided by the authors to give readers additional information about their work.

Supplement to: Ruiling Feng, Xian Xiao et al.
Metabolic Impacts of Low-Dose IL-2 Therapy in Sjögren’s Syndrome: Insights from a Post Hoc Analysis of a Clinical Trial

Table of Contents

1. Supplementary Tables.. 2

Supplementary Table 1. Monoclonal antibodies used in flow cytometric analysis in human…...…………………………….. 3

Supplementary Table 2. Altered serum metabolites after low-dose IL-2 therapy……………………………………………....................................... 4

Supplementary Table 3. Changes of metabolic pathways related to low-dose IL-2 therapy………………………………….. 5

Supplementary Table 1. Monoclonal antibodies used in flow cytometric analysis in human

Antigen	Clone	Fluorochrome	Vendor
CD3	OKT3	Brilliant Violet 650	Biolegend
CD4	RPA-T4	Alexa Fluor 700	Biolegend
CD45RA	HI100	APC-Cy7	Biolegend
CD25	M-A251	PE-CF594	BD Biosciences
CD127	A019D5	Brilliant Violet 605	Biolegend
PD-1	EH12.2H7	Brilliant Violet 711	Biolegend
CXCR5	RF8B2	Alexa Fluor 647	Biolegend
CCR7	G043H7	PE-Cy7	Biolegend
CXCR3	G025H7	Brilliant Violet 421	eBioscience
CCR4	TG6	PerCP-Cy5.5	Biolegend
CCR6	G034E3	PE	Biolegend

Supplementary Table 2. Altered serum metabolites after low-dose IL-2 therapy

Metabolites	Change	Proposed mechanism of gut microbiota modulation	Reference
Acetyl-CoA	↑	A central metabolic intermediate, influence the activity or specificity of multiple enzymes，reflects the general energetic state of the cell.	Pietrocola at al. Cell Metab. 2015
Ascorbic acid	↑	Increase anti-inflammatory, antiviral and antibacterial function in both innate and adaptive immune system.<br>Promotes the oxidant scavenging activity against environmental oxidative stress.	Carr at al. Nutrients. 2017
Citrate	↑	In normal medium, citrate inhibited LPS-induced tumour necrosis factor (TNF)-α and interleukin (IL)-8 transcripts, inhibit pro-inflammatory cytokine production via modulation of inflammatory gene transactivation.	Ashbrook et al. Clin Exp Immunol. 2015
Buyryl-Coa	↑	Influence butyrate production.<br>Important within the colonic microbial community.<br>Impact on Tissue Acyl-CoA and Histone Acetylation Levels.	Louis et al. Environ Microbiol. 2010
Glutathione	↑	Responsible for the detoxification of reactive oxygen and nitrogen species (ROS/RNS) and electrophiles produced by xenobiotics.<br>Tregs appear to block GSH redistribution from the nucleus to the cytoplasm in Teffs , than suppresse of T cell activation and proliferation.	Morris et al. Mol Neurobiol. 2014<br>Yan et al. J Biol Chem. 2010
Glycoursodeox-ycholic acid	↑	The ligand GPBAR1 and FXR) expressed on T, dendritic cells and natural killer T cells and might join the immune and metabolic disorders.	Fiorucci et al. Front Immunol. 2018
Acetic acid	↑	The microbial metabolites, short-chain fatty acids, regulate colonic Treg cell homeostasis.	Smith et al. Science. 2013
Isoleucine	↓	Induces expression of defense peptides (adaptive immunity. i.e., β-defensins) that can regulate host innate and adaptive immunity.	Gu C et al. Curr. Protein Pept. Sci. 2019
Glyceraldehyde 3-phosphate dehydrogenase	↓	NADPH down-regulates aerobic glycolysis in activated myeloid and lymphoid cells, which mediates its anti-inflammatory effects.	Kornberg MD, et al. Science, 2018
Urea	↓	Activates TLR1 and TLR2 then stimulates the NF-κB activation to trigger the downstream cytokines, such as TNF-α and secreted alkaline phosphatase (SEAP).	Chen et al. Eur J Med Chem. 2019

Supplementary Table 3. Changes of metabolic pathways related to low-dose IL-2 therapy

Metabolites pathway	Proposed mechanism of serum metabolites modulation	Reference
Phenylacetate metabolism	Phenylacetate acetate induces DNA synthesis, IL-2 production, IL-2receptor a (CD25) and B-chain (CD122) expression in human PBMC.	Sudeepta et al. Journal of Clinical Immunology,1994
Ubiquinone biosynthesis	plays a pivotal role in OXPHOS, which supports the proliferation of T cells. Esp. effector and regulatory T cells .	Martin et al. Science signaling, 2015.
Pyruvate metabolism	Pyruvate overload of the mitochondria increases ROS production, and this oxidative modification of pyruvate kinase enables this cytoplasmic enzyme to enter the nucleus, phosphorylate STAT3 and promote IL-6 production.	Cornelia et al. Current Opinion in immunology, 2017
Glutamate metabolism	Blocking Th17 cells and promotes iTreg cells.	Tao et al. Nature,2017
Urea cycle	Activates TLR1 and TLR2 then stimulates the NF-κB activation to trigger the downstream cytokines, such as TNF-α and secreted alkaline phosphatase (SEAP).	Chen et al. Eur J Med Chem. 2019
CoA biosynthesis	Play an important role in IL-4- mediated macrophage polarization.	Ajit et al. Cell Metab. 2018
